# Spatial and temporal scales of aquatic bacterial beta diversity

**DOI:** 10.3389/fmicb.2012.00318

**Published:** 2012-08-31

**Authors:** Stuart E. Jones, Tracey A. Cadkin, Ryan J. Newton, Katherine D. McMahon

**Affiliations:** ^1^Department of Biological Sciences, University of Notre DameNotre Dame, IN, USA; ^2^Department of Civil and Environmental Engineering, University of Wisconsin-MadisonMadison, WI, USA; ^3^School of Freshwater Sciences, Great Lakes WATER Institute, University of Wisconsin-MilwaukeeMilwaukee, WI, USA; ^4^Microbiology Doctoral Training Program, University of Wisconsin-MadisonMadison, WI, USA

**Keywords:** lake, beta diversity, spatial, temporal

## Abstract

Understanding characteristic variation in aquatic bacterial community composition (BCC) across space and time can inform us about processes driving community assembly and the ability of bacterial communities to respond to perturbations. In this study, we synthesize BCC data from north temperate lakes to evaluate our current understanding of how BCC varies across multiple scales in time and space. A hierarchy of average similarity emerged with the highest similarity found among samples collected within the same lake, especially within the same basin, followed by similarity among samples collected through time within the same lake, and finally similarity among samples collected from different lakes. Using decay of similarity across time and space, we identified equivalent temporal (1 day) and spatial (10 m) scales of BCC variation. Finally, we identify an intriguing pattern of contrasting patterns of intra- and inter-annual BCC variation in two lakes. We argue our synthesis of spatio-temporal variation of aquatic BCC informs expectations for the response of aquatic bacterial communities to perturbation and environmental change. However, further long-term temporal observations will be needed to develop a general understanding of inter-annual BCC variation and our ability to use aquatic BCC as a sensitive metric of environmental change.

## Introduction

A central goal of ecology is to understand the patterns and processes of biodiversity. In particular, community ecologists seek to describe species richness at local scales (alpha diversity), differences in diversity across space and time (beta diversity), and diversity within a region (gamma diversity). Following the emergence of molecular techniques to classify bacteria, the field of freshwater microbial ecology has generated a number of studies describing the extant alpha diversity in these systems (Zwart et al., 2002; Newton et al., 2011). Like many ecologists (Soininen, 2010; Anderson et al., 2011), researchers studying microbial diversity are now transitioning to focus more intently on beta diversity (i.e., species turnover) in an effort to identify both extrinsic and intrinsic factors that explain differences in taxon composition among communities separated in space or time.

Distinct and consistent taxon distribution patterns along gradients in space and time are emerging (Schauer et al., 2005; Wu et al., 2006; Newton et al., 2007, 2011; Jones et al., 2009; Jezberova et al., 2010; Simek et al., 2010; Eiler et al., 2012). For example, repeatable compositional responses to predation (Pernthaler, 2005), interactions with phytoplankton (Pinhassi et al., 2004; Kent et al., 2007) and chemical or resource gradients (Schauer et al., 2005; Wu and Hahn, 2006a; Newton et al., 2007; Jones et al., 2009) have been observed. Many of the initial efforts to quantify aquatic microbial beta diversity have focused on traditional biogeographical concepts, including taxa-area relationships (Reche et al., 2005; Lindstrom et al., 2007; Logue et al., 2011) and the niche vs. neutral debate (Langenheder and Ragnarsson, 2007; Jones and McMahon, 2009; Lindstrom et al., 2010). Despite numerous studies addressing these biogeographical concepts, we, as aquatic microbial ecologists, lack a basic understanding of the characteristic scales of variation in aquatic bacterial community composition (BCC) (Lindstrom and Langenheder, 2012); this is especially true for variation in time. Arguably, this is a key gap in our basic understanding of aquatic bacterial diversity that hinders our ability to develop theories about how microbial mediated function and the stability of those functions are maintained across space and time.

Freshwater microbial ecologists are not alone in their plight to understand the characteristic temporal and spatial scales of microbial community beta diversity. Recent work in the marine environment and among human body sites has revealed systematic variation in bacterial community similarity with distance and time (Fuhrman et al., 2006; Caporaso et al., 2011; Gilbert et al., 2012). Human microbiome research has identified bacteria that are endemic to a particular body site and temporal decay of community similarity with time at a given body site (Caporaso et al., 2011). Work in the marine environment has also shown distance decay relationships and cyclic, seasonal patterns in the similarity of bacterial communities at a given site (Fuhrman et al., 2006; Gilbert et al., 2012). Despite widespread patterns in spatial and temporal decay of bacterial community similarity across ecosystems, we lack any mechanistic understanding of the underlying processes driving these microbial biogeographic and temporal patterns (Hanson et al., 2012). We argue that an understanding of the temporal and spatial scales over which these patterns occur will be indicative of underlying process.

In this study, we use a collection of unpublished and previously published datasets from north temperate lakes to explore patterns of variation in freshwater BCC across multiple temporal and spatial scales. We employed a “distance-decay approach” (Soininen et al., 2007) to examine species turnover among samples. Our questions included: (1) how variable are PCR-based measures of community composition and does this limit our ability to quantify microbial beta diversity? (2) How does within-lake compositional variation compare to across-lake compositional variation? and (3) At multiple scales, how does spatial compositional variation compare to temporal compositional variation?

## Materials and methods

### Study sites and sample collection

Lake Mendota (ME) and Crystal Bog (CB) Lake (both in Wisconsin, USA; Table 1) were each sampled at 32 sites to evaluate the BCC within the lakes at a relatively high spatial resolution, on June 18, 2007. Sampling of both lakes was conducted on the same day by two separate research teams. In the smaller CB, approximately 5 min was spent at each site and all 32 sites were sampled in approximately 4 h. Sampling of the larger ME occurred over 6 h, with approximately 5 min spent at each site. ME and CB samples were filtered in the lab after being stored on ice in a dark cooler during the field sampling effort. Hold times in the field prior to filtering varied between 0.5 and 5.5 h. Sampling locations were determined based on a rectangular, uniform grid that was fit over a map of the lake in such a way that 32 grid cells (15.5 m by 11.5 m in CB and 1000 m by 1300 m in ME) encompassed the entirety of the lake surface. For each grid cell, a latitude and longitude was randomly selected to determine the sampling point. At each location, three integrated samples of the top 1 m of the water column (representing the epilimnion) were collected, transferred to 1-L bottles, and stored on ice. A 200-mL subsample was vacuum filtered onto a 0.2-μ m filter (Pall Life Sciences) and stored at −80°C until DNA was extracted.

**Table 1:** **Characteristics of Lake Mendota (ME) and Crystal Bog (CB) Lake**.

**Feature**	**Mendota**	**Crystal Bog**
Location	46.01°N, 89.61°W	43.10°N, 89.41°W
Surface area (ha)	3938	0.5
Mean depth (m)	12.8	1.7
Max depth (m)	25.3	2.5
Trophic status	eutrophic	dystrophic
Shoreline development	high	low
Total phosphorus (μg L^-1^)	109.5	18.2
pH	8.4	5.2
DOC (mg L^-1^)	5	9.8
Conductivity (μS m^-1^)	412	11
Wind speed (ms^-1^)	4.7	1.0

We present long-term averages from routine sampling by the NTL-LTER (1981–2010 for CB and 1995–2010 for ME). Mean wind speed data are from high-resolution data collected 2005–2011 for both lakes.

In addition to our highly resolved spatial survey, 62 total integrated-epilimnion samples were collected across three open-water seasons (2003, 2005, and 2007) from the center of CB, as previously described (Kent et al., 2004). Exact dates of sample collection can be found in Supplementary Material.

### DNA extraction and automated ribosomal intergenic spacer analysis (ARISA)

DNA was extracted from each of the filters using the FastDNA kit using the manufacturer's protocol (QBiogene) and stored at −80°C until needed. The DNA was quantified using Picogreen (Molecular Probes) and a Molecular Devices Spectramax fluorometric-capable plate reader. Samples were diluted with sterile water in order to add 5–10 ng of template DNA for each ARISA PCR reaction. Samples were then amplified (Eppendorf Mastercycler) using the 1406F fluorescently labeled primer (5′-TGYACACACCGCCCGT-3′) and the 23SR primer (5′-GGGTTBCCCCATTCRG-3′; bacterium specific, 23S rRNA gene) according to the following conditions: 2 min at 94°C, followed by 30 cycles of 94°C for 35 s, 55°C for 45 s, and 72°C for 2 min, then finishing with 72°C for 2 min. After amplification, the samples were mixed with a formamide buffer and a 100–2000-bp custom internal size standard (Bioventures) before denaturing capillary electrophoresis was carried out on an ABI 3730 genetic analyzer (PE Biosystems). For pictoral examples of ARISA profiles see Fisher and Triplett (Fisher and Triplett, 1999). Electropherograms were analyzed using custom fragment analysis utilities developed in the R Statistics Environment (Jones and McMahon, 2009). Briefly, community profiles were de-noised, individual peaks were binned into operational taxonomic units (OTUs), and the presence or absence of each OTU in a profile was based upon peak presence or absence in that profile. In general, ARISA can resolve OTUs with a single bp difference in length when fragments are 300–1000 bp in length. As lengths increase from 1000 to 1500 bp (the maximum length considered) resolution decreases to 3–5 bp due to smearing or stretching of bands migrating through the sequencer capillary. The width of OTU bins is adjusted as a function of length to accommodate this change in resolution. Further details of the ARISA method can be found elsewhere (Fisher and Triplett, 1999; Brown et al., 2005; Jones et al., 2007; Jones and McMahon, 2009). This resulted in sample-by-OTU-presence-absence matrices for each sample set that was analyzed statistically, as described below.

### Methodological variation

In order to explore variation in BCC across time and space, we must understand the repeatability of our methods. To this end, we quantified the error in all steps of ARISA by sequentially replicating DNA extraction, ARISA PCR, and capillary gel electrophoresis for a single sample from ME, WI collected on July 17th, 2007. We filtered 250 ml of ME water collected from the top 1 m of the water column, onto each of four filters. We then extracted DNA from these filters with the methods described above. DNA from each of the four extractions was used as template in four replicate ARISA PCR reactions. Finally, we ran four replicate fragment analysis capillaries from each PCR reaction (yielding a total of 64 ARISA profiles from a single lake sample).

### Previously published north temperate lakes microbial observatory (NTL-MO) datasets

The collection of studies conducted by the North Temperate Lakes Microbial Observatory (NTL-MO) over the past decade represent a set of consistently collected and treated (both molecularly and analytically) data spanning broad ranges in space and time. All samples were derived from the integrated epilimnion water column. ARISA data from three years (2000, 2001, and 2005) of a multi-year temporal survey (36 samples from a single location in ME) (Shade et al., 2007), a purely spatial survey (90 samples from 13 Wisconsin lakes) (Yannarell and Triplett, 2004), and a combined spatial/seasonal survey (90 samples from 30 Wisconsin lakes sampled in June, July, and October) (Yannarell and Triplett, 2005) were accessed from the NTL-MO database (http://microbes.limnology.wisc.edu/) and used to supplement the previously unpublished data described above. Methods for collection, molecular analyses, and electropherogram interpretation can be found in the original publications. All samples were collected during the open-water season. Table 2 contains the total number of samples used from each study and the spatio-temporal extent of the datasets. A complete list of samples can be found in Supplementary Material.

**Table 2:** **Studies and number of samples included in the comparison of spatial and temporal scales of aquatic bacterial community compositional similarity**.

**Study**	**Site**	**Basin**	**<1 week**	**<1 month**	**<1 year**	**>1 year**	**Cross-lake**
Shade et al., 2007							
Yannarell and Triplett, 2004							
Yannarell and Triplett, 2005							
This study							
Total number of samples	282	333	114	114	204	114	372

Black cells indicate the spatial and temporal scales considered in the study.

### Statistical approaches

To assess the extent of variation introduced by our data collection methods, we compared the 64 ARISA profiles collected from a single sample from ME using minimum and mean similarities across replicates and analysis of similarity (ANOSIM) from the *vegan* package in the R Statistics Environment (R Development Core Team, 2010).

To assess beta diversity (i.e., species turnover), we used multivariate similarities (Sørensen's Index; $\frac{2C}{A+B}$, where *C* is the number of species shared between the two samples and *A* and *B* are the richness of each sample; Legendre and Legendre, 1998) amongst a set of samples as our response variable. Using this standardized metric of compositional variability allowed for comparison across studies despite slight differences in OTU bin definitions.

The Sørensen's similarity matrices from our single day, high spatial-resolution sampling, were used to create Principle Coordinate Analysis (PCoA) ordinations. Mean centroids of triplicate samples taken at each of the 32 sites were used for display. Simultaneous display of compositional similarity and geographic location of samples is challenging. To depict this information, we assigned gradients of color to the first two axes of our PCoA ordinations and therefore the color of a location on the lake map is indicative of the composition of the community at that location. Geographic locations with similar colors on the map had similar BCC. The spatial interpolation and plotting of the compositional data were conducted using the *spatstat* package (Baddeley and Turner, 2005) in the R Statistics Environment (R Development Core Team, 2010).

In an attempt to compare rates of change in BCC through time and space, we fit distance-decay relationships for all of our data. Before estimating the distance-decay relationship, both the predictors (time or geographic distance) and Sørensen's similarity values were log transformed after adding a small value (1 day/meter or 0.001 for Sørensen's indices) to avoid log of zero issues, as is traditionally done when estimating the decay of community similarity over space or time (Soininen et al., 2007). When considering decay in community similarity over time, documented differences in inter-annual variation in CB (each year is distinct from previous; Kent et al., 2004) and ME (repeated annual phenology has been observed; Shade et al., 2007) forced us to use continuous time and julian day-based time, respectively, for the two time series. We followed the approach of Soininen and colleagues (2007) to calculate the predicted compositional similarity between samples separated by one meter or one day and the distance or time between samples required to halve the compositional similarity. We also used *T*-tests to identify significant differences in mean Sørensen's similarity between groups of samples.

When using pair-wise similarity scores for both the distance-decay model fitting and *T*-tests issues of non-independence are encountered. To avoid these non-independence issues, we used a randomization technique for assessing the significance of our distance-decay relationship (Green et al., 2004; Horner-Devine et al., 2004). For our *T*-tests we used two different approaches depending on the nature of the data. If we were comparing similarities within a single pair-wise similarity matrix we used a sub-sampling-based, pair-wise-adjusted *T*-test (paT) approach as described by Danforth and Freeman-Gallant (1996). When comparing similarities from multiple studies and therefore from different pair-wise similarity matrices we used the Pooled Mean Diversities test outlined by Gilbert, Rossini, and Shankarappa (GRStest; Gilbert et al., 2005). Briefly, the test estimates the mean pair-wise difference for two groups of samples as the empirical mean, but uses a variance estimate that takes into account non-independence of pair-wise comparisons through the use of U-statistic theory. All statistical analyses were conducted using custom created functions in the R Statistics Environment (R Development Core Team, 2010).

## Results

### Quantification of methodological variability

The nested replication of the ARISA procedure allowed us to quantify and partition methodologically induced variability. The minimum Sørensen's similarity across the entire set of 64 analyses from a single sample was 0.92, and the mean was 0.95. The minimum and mean Sørensen's similarity of profiles generated from a single DNA extraction was 0.94 and 0.97, respectively. ARISA profiles significantly clustered by extraction, and differences among replicate PCR reactions carried out on a single DNA extraction were also detected, but as indicated by overall mean similarity across all profiles, these differences were small (Table 3). The minimum and mean similarity across capillary runs was 0.98, and no difference was detected between replicate capillary runs conducted on a single PCR reaction (Table 3). Overall, methodological variation was small relative to differences observed across space and time.

**Table 3:** **Minimum and mean Sørensen's similarity of replicate profiles at three major steps of the ARISA procedure**.

**Portion of method**	**Minimum similarity**	**Mean similarity**	**ANOSIM R**	***p*-value**
Water filtering and DNA extraction	0.92	0.95	0.88	0.001
PCR on a single DNA extraction	0.94	0.97	0.81	0.001
Capillary run on a single PCR reaction	0.98	0.98	−0.17	1.0

In addition, statistics for analysis of similarity (ANOSIM) comparisons run across replicates at each step of the ARISA procedure.

### Within-lake vs. across-lake spatial heterogeneity

**Figure 1:**
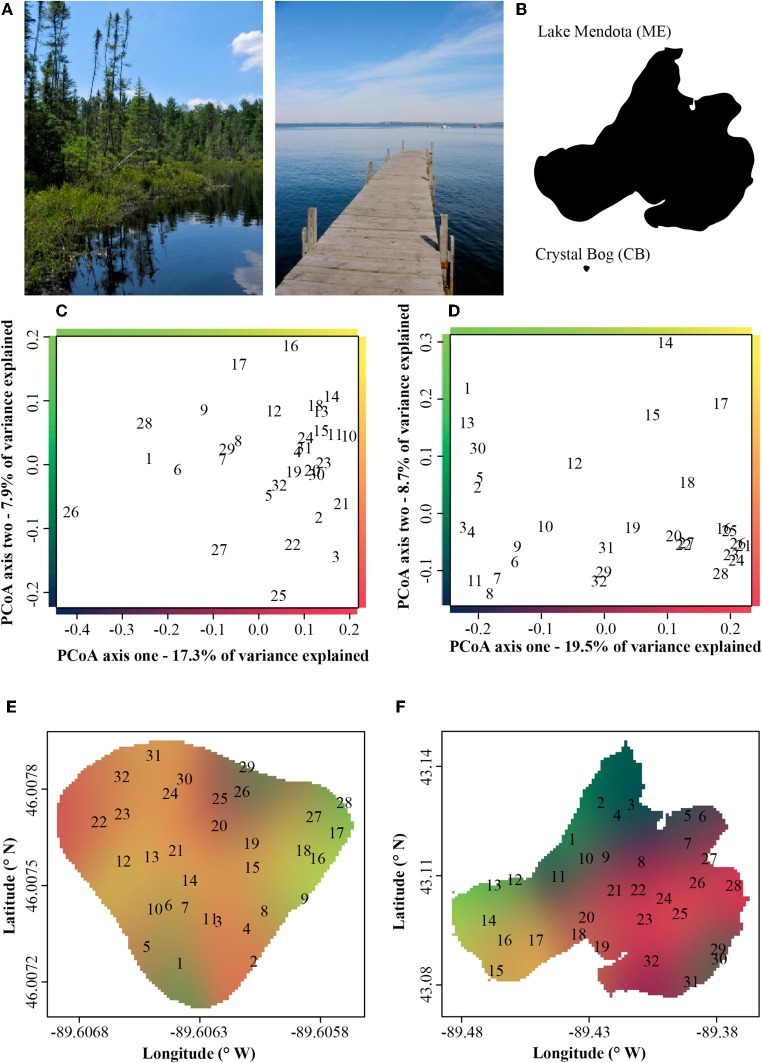
**(A)** Pictorial and **(B)** areal contrast in the Lake Mendota (ME) and Crystal Bog (CB) environments. Principle coordinate analysis (PCoA) ordinations of bacterial community composition at 32 sites in Crystal Bog **(C)** and Lake Mendota **(D)**, collected on a single day. Points indicated as sample site numbers represent the mean of triplicate ordinated samples. The axes of the ordinations are color-coded and these colors are used to indicate bacterial community composition on the lake maps in panels **E** (Crystal Bog) and **F** (Lake Mendota).

**Figure 2:**
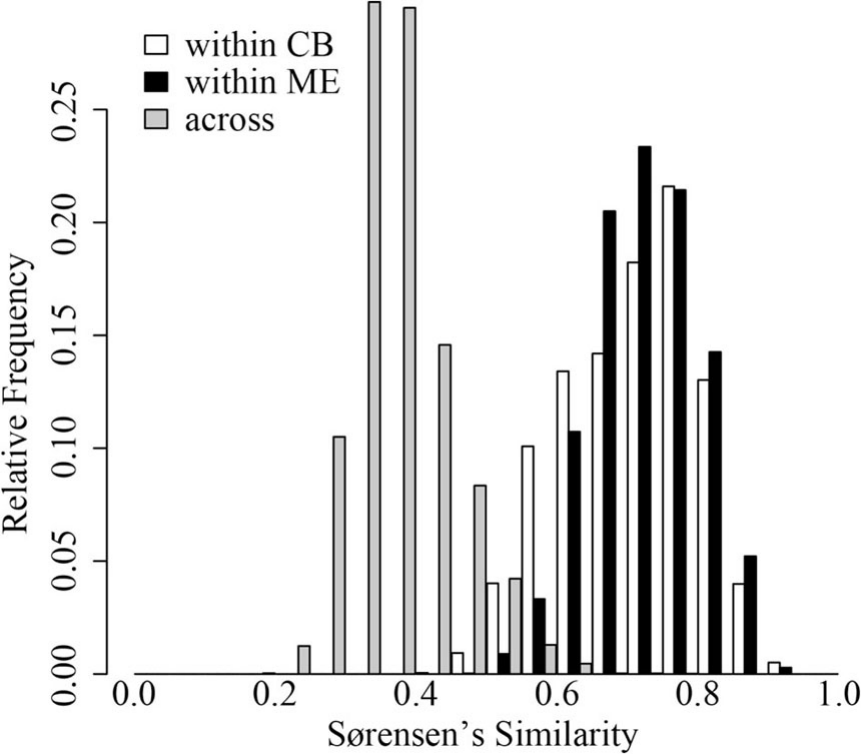
**Distribution of Sørensen's similarity indices within 32 sites in Crystal Bog (within CB; open bars), within 32 sites in Lake Mendota (within ME; black bars), and between sites in Crystal Bog and Lake Mendota (across lakes; gray bars)**. Similarities were calculated as pair-wise comparisons of all samples within a set (e.g., 512 pairwise combinations for the 32 sites in CB).

In both ME and CB we observed substantial horizontal variation in BCC on our single date of collection (Figure 1). In both lakes, triplicate samples collected within a 1-m^2^ site were significantly more similar than cross-site (within-lake) comparisons (paT, CB: *df* = 74, *t* = 2.57, *p* < 0.05; ME: *df* = 75, *t* = 8.61, *p* < 0.01). Site-to-site differences were greater in ME, as demonstrated by comparison of *t*-values for the above paT (2.57 and 8.61 for CB and ME, respectively), but the magnitude of greatest differences in each lake were similar (Figure 2).

### Spatial vs. temporal compositional variability

**Figure 3:**
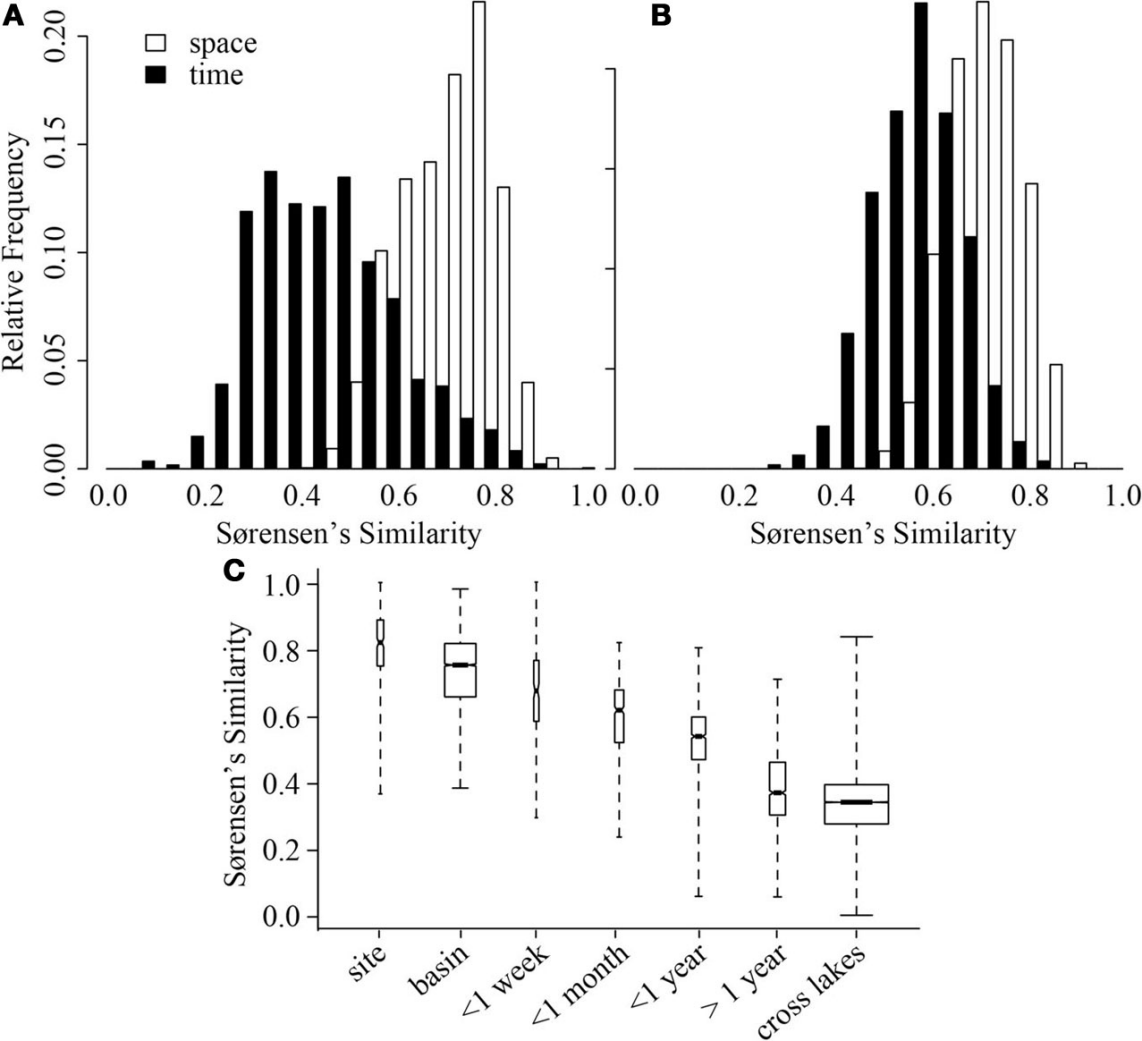
**Distribution of Sørensen's similarity indices for samples from Crystal Bog (A) and Lake Mendota (B) distributed spatially within the lake (open bars) and through time (black bars)**. Comparisons across space were conducted using the 32 samples collected on a single day from each lake. Comparisons across time were conducted using 68 samples from CB collected in 2003, 2005, and 2007 (previously unpublished), and 46 samples from ME collected in 2000, 2001, and 2005 (Shade et al., 2007). **(C)** Distribution of pairwise Sørensen's similarities calculated for multiple scales of time and space. Bold lines delineate medians, box heights represent the interquartile range, and whiskers mark the extremes of the data. Width of a box is proportional to the number of similarities in the category. Data from: Yannarell and Triplett (2004, 2005); Shade et al. (2007), and the current study, see Table 2.

In both ME and CB, spatial variation at a single time point was significantly smaller than variation through time (GRStest—CB: *T* = 17.8, *p* < 0.001; ME: *T* = 11.3, *p* < 0.001), but average spatial similarity across lakes was smaller than similarity within a lake through time (Figures 3A,B). However, the ranges of similarities observed through time and across lakes were approximately the same. When more lakes were considered, a hierarchy of average similarity emerged with the highest similarity found among samples collected within the same lake, especially within the same basin, followed by similarity among samples collected through time within the same lake, and finally similarity among samples collected from different lakes (Figure 3C).

A comparison between variation in time and space can be made using the decay of compositional similarity with distance and time. We observed significant log-log relationships between Sørensen's similarity decay over distance (*R*^2^ = 0.25, *p* < 0.001) and time (*R*^2^ = 0.39, *p* < 0.001) (Table 4). Comparison of estimated similarity of two communities separated by a single meter or day (0.90 vs. 0.84) and the halving distance of community similarity across distance or time (~4000 m vs. ~350 days) suggests that one day of temporal separation is approximately equivalent to 10 m of physical separation in terms of community change or turnover.

**Table 4:** **Summary statistics for distance- and temporal-decay relationships fit to our bacterial community similarity data from north temperate lakes**.

	**Slope^a^**	**Intercept^b^**	**Similarity^c^ when separated by 1 meter or 1 day**	**Halving distance (m) or time (day) of similarity^c^**
Distance decay	−0.084	−0.046	0.90	4000
Temporal decay	−0.118	−0.076	0.84	350

a Slope of the distance-decay linear relationship fit to log transformed Sørensen's similarity values.

b Intercept of the distance-decay linear relationship fit to log transformed Sørensen's similarity values.

c Predicted similarity, halving-distance, and halving-time were predicted using the methods of Soininen and colleagues (2007).

### A contrast in inter-annual community compositional patterns

**Figure 4:**
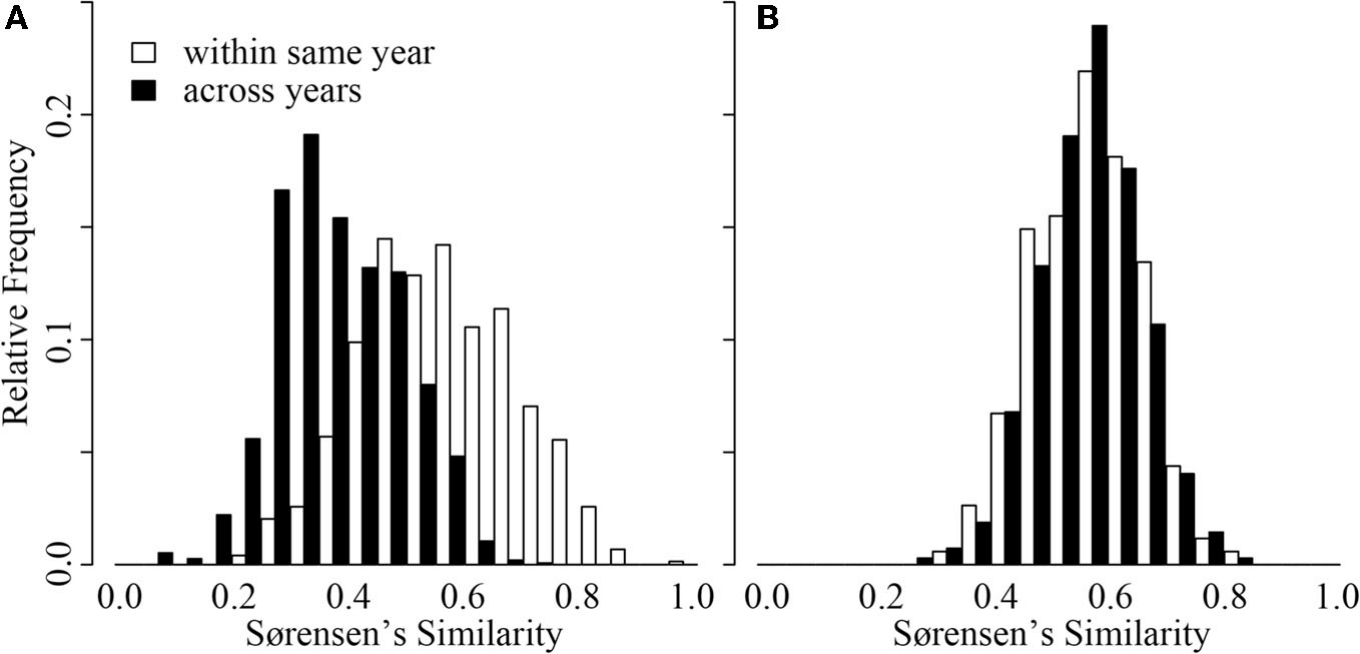
**Distribution of Sørensen's similarity indices for samples collected within the same year (open bars) and samples collected across years (black bars) from Crystal Bog (A) and Lake Mendota (B)**. Comparisons were conducted using 68 samples from CB collected in 2003, 2005, and 2007 (previously unpublished), and 46 samples from ME collected in 2000, 2001, and 2005 (Shade et al., 2007).

Inter-annual patterns of change in CB were distinct from those in ME (Figure 4). Although intra-annual variation was similar in magnitude across the two lakes (GRStest: *T* = −1.16, *p* > 0.1), CB had much greater inter-annual variation (GRStest: *T* = −18.7, *p* < 0.001). In fact, CB inter-annual similarity was significantly smaller than intra-annual similarity (paT: *df* = 64, *t* = 9.18, *p* < 0.01). This was not the case for ME (paT: *df* = 42, *t* = −0.12, *p* > 0.1). Effectively, ME BCC varied as much within a single year as it varied across any given year, suggesting a repeated annual phenology as previously documented by Shade and coauthors (Shade et al., 2007). Alternatively, CB BCC across years was more variable, suggesting directional change from year to year with smaller scale variation within a given year (Figure 4).

## Discussion

The goal of our study was to calibrate expectations for beta diversity of aquatic lake bacterial communities across multiple spatial and temporal scales. The majority of spatial surveys have focused on cross-lake comparisons, and those that do consider within-lake variations generally do not consider it in a continuous manner (but see Yannarell and Triplett, 2004). Temporal studies are rarer than spatial lake surveys, but time appears to be an equally important axis of variation in aquatic microbial communities. Therefore, we sought to compare spatial and temporal beta diversity in order to evaluate their relative importance in structuring microbial communities.

As BCC is compared across smaller and smaller spatial and temporal scales, we may approach the limits of our methods to discriminate assemblages. To identify the limitations of our methods we conducted a nested evaluation of our selected method to quantify microbial beta diversity (ARISA; Table 3). The ARISA method proved to be extremely repeatable. Our results suggest that we can detect compositional differences, as quantified by Sørensen's Index, of approximately 0.05 or greater. The nested-nature of our method evaluation indicated that DNA extraction generates the greatest variation, followed by PCR, while the capillary gel electrophoresis and analysis imparts nearly no methodological variability. Similar evaluation should be conducted for newer molecular methods being used to characterize microbial beta diversity (e.g., tag pyrosequencing; Prosser, 2010).

It should be noted that ARISA, as well as any other fingerprinting technique or even low coverage tag pyrosequencing, are biased toward the detection of abundant community members. As a result, our analyses neglect the contribution of likely numerous rare members of aquatic bacterial communities (Pedros-Alio, 2006). It is difficult to predict how the inclusion of the rarer members of aquatic bacterial communities would impact the patterns we observe here. On one hand, if these rare members are extremely endemic spatial differences may be magnified, while temporal changes may be muted. On the other hand, if the rarer bacterial community members represent a relatively homogeneous, seed bank (Jones and Lennon, 2010; Caporaso et al., 2012) across the landscape, spatial and temporal differences may be reduced. It does, however, seem that the use of a consistent sampling depth in all systems generates a comparable rate of community similarity change with time or distance where increasing the sampling depth only impacts the intercept of this relationship (Horner-Devine et al., 2004).

Spatial surveys have been a popular approach to characterize freshwater bacterial diversity and structuring features of aquatic BCC. Broad spatial surveys have identified stark contrasts in occurrence patterns of some freshwater bacterial taxa (Lindstrom et al., 2005; Yannarell and Triplett, 2005; Wu et al., 2006; Newton et al., 2007; Simek et al., 2010). Phylogenetically narrow freshwater lineages appear to respond strongly to environmental variables, including pH (Newton et al., 2007; Simek et al., 2010), carbon substrate characteristics (Jones et al., 2009; Salcher et al., 2011), temperature (Wu and Hahn, 2006b), and salinity (Wu et al., 2006). Indeed, environmental characteristics seem to be key in determining BCC of aquatic ecosystems (Lindstrom et al., 2005; Yannarell and Triplett, 2005; Berdjeb et al., 2011) and dispersal limitation or biogeography seems to be less important in spatial structuring of aquatic bacterial communities (Crump et al., 2007; Van der Gucht et al., 2007; Jones and McMahon, 2009; Nelson et al., 2009). However, robust biogeographic patterns, such as distance-decay of community similarity and taxa-area relationships, have been observed in aquatic microbes (Soininen et al., 2011) and other microbial systems (Green et al., 2004; Horner-Devine et al., 2004; Martiny et al., 2006) indicating some relationship between geographic distance and likelihood of dispersal in microbial communities.

Although freshwater bacterial biogeographic patterns have been considered previously, only a few prior studies have considered intra-lake differences (Yannarell and Triplett, 2004; De Wever et al., 2005). Some might argue that lakes are horizontally well-mixed and one should expect to see very little intra-lake heterogeneity in the x-y dimensions. Under this premise, we would expect a flat distance-decay slope until spatial scales included comparisons of communities across lake boundaries. The results of our highly resolved intra-lake spatial survey suggest that within-lake x-y spatial heterogeneity in BCC does indeed exist (Figure 1). In addition, we did not observe any habitat-specific patterns of community composition. For example, there was not a consistent contrast in composition between littoral and pelagic sites. Instead, we observed a consistent decay in community similarity from spatial scales of meters to hundreds of kilometers (Figure 3C). In fact, the halving distance of community similarity is surprisingly similar to that observed by Soininen et al. (2011), who only evaluated cross-lake community similarity and geographic distances [4000 m in our study vs. 2965 m in Soininen et al. (2011)]. As a result of similar slopes, the initial similarities observed in our study at a distance of 1 m (0.9) were much higher than the 0.5 observed by Soininen and colleagues (2011).

Surprisingly, we observed comparable levels of beta diversity in the two lakes sampled in our intense spatial survey (Figure 2). Based upon previous work (Yannarell and Triplett, 2004), we expected a greater level of beta diversity in the larger lake. Our results indicate that even very small lakes (~1 ha) can have significant horizontal spatial beta diversity (Figure 2B). The presence of horizontal heterogeneity in community composition may indicate the rates of biological and ecological interactions driving bacterial community assembly are occurring more rapidly than rates of water movement and turbulence in lakes; this heterogeneity may include neutral dynamics occurring in temporarily separated parcels of water.

Although long-term or well-resolved temporal surveys targeting aquatic bacterial communities are fairly rare, some progress has been made toward understanding key factors influencing BCC in freshwater habitats through time (Kent et al., 2004; Allgaier and Grossart, 2006; Wu and Hahn, 2006a; Shade et al., 2007; Crump et al., 2009). Successional or phenological patterns have also been linked to changes in the physical and chemical environment (Kritzberg et al., 2006; Wu and Hahn, 2006a; Shade et al., 2007; Nelson, 2009) as well as changes in other components of the microbial loop (Muylaert et al., 2002; Kent et al., 2004; Wu and Hahn, 2006a). Temporal analogs of the approaches used to investigate spatial beta diversity have been developed, including the species-time relationship (Preston, 1960; Adler and Lauenroth, 2003; White, 2004) and temporal decay of compositional similarity over time (Collins et al., 2000; Korhonen et al., 2010). Using these tools we can compare the strength of temporal and spatial effects on freshwater bacterial beta diversity.

Our results suggest that spatial variation and temporal variation are quite comparable (Figure 3; Table 4). For example, the average community similarity across multiple temporal scales falls between average intra- and inter-lake community similarity values and there is a large amount of overlap in the distribution of these values (Figure 3). Using the temporal and spatial similarity decay relationships, we were able to calibrate temporal and spatial beta diversity to each other. Communities separated by a single day or meter are comparably similar, and we expect compositional similarity to halve across approximately one year or 4000 m (Table 4; Soininen, 2010). The equivalence of a day and a few meters in their impact on bacterial community similarity suggests similar ecological processes driving community assembly occur over these scales in space and time (Soininen, 2010). Soininen (2010) highlighted intrinsic factors, such as body size and dispersal rate, and extrinsic factors, such as ecosystem size and isolation, as likely drivers of bacterial turnover in both space and time. We agree with this theoretical assessment and suggest the results of our study support this assertion. Perhaps the change in environmental characteristics that occur over a day is equivalent to aquatic spatial heterogeneity occurring on the scale of meters. Alternatively, aquatic bacteria generation times (approximately on the order of days) may closely correspond to aquatic bacterial dispersal distances or rates. As has been highlighted recently, a shift in focus to the processes underlying current microbial biogeographic and temporal observation is now required (Hanson et al., 2012), and our results may indicate at what spatial and temporal scales to begin investigation of underlying processes, such as competitive exclusion, dispersal, and neutral drift in community composition.

An additional intriguing temporal observation from our study was the contrast in inter-annual patterns between CB and ME (Figure 4). Despite comparable spatial beta diversity, our analysis (Figure 4) and previous work (Kent et al., 2004) suggest that repeated seasonal patterns in BCC do not occur in CB, while extremely repeatable phenological patterns occur in ME each year (Figure 4; Shade et al., 2007). This represents empirical support for hypothetical patterns describing temporal decay of community similarity in seasonal and non-seasonal communities presented by Korhonen et al. (2010). However, we are uncertain what could drive this contrast in dynamics. ME and CB differ in a number of characteristics, including trophic status, lake size, pH, and surrounding land use, making it difficult to identify what system features drive this divergence. We emphasize the need for larger datasets of intra- vs. inter-annual variation in BCC in multiple lakes representing various gradients (e.g., size, trophic status) to develop more robust expectations.

### Conflict of interest statement

The authors declare that the research was conducted in the absence of any commercial or financial relationships that could be construed as a potential conflict of interest.

## Figures and Tables

**Table S1:** **Collection details for all samples included in this study**. All samples were integrated samples extending from the lake surface to the indicated depth. NA indicates the information was unavailable.
